# Development and application of a RAA-CRISPR/Cas12a-based detection system for the pseudorabies virus *gE* gene

**DOI:** 10.3389/fmicb.2025.1641525

**Published:** 2025-09-16

**Authors:** Chunlian Song, Jiajia Wei, Song Du, Yalong Sun, Chen Guan, Jianqin Li, Qianfei Wei, Xue Zhang, Hong Shen, Xianghua Shu

**Affiliations:** ^1^College of Veterinary Medicine of Yunnan Agricultural University, Kunming, China; ^2^College of Animal Science and Technology of Yunnan Agricultural University, Kunming, China

**Keywords:** pseudorabies virus, CRISPR-Cas12a, recombinant-aided amplification, veterinary diagnostics, point-of-care diagnostics, molecular diagnostics

## Abstract

The development of field-deployable diagnostic tools for pseudorabies virus (PRV) surveillance remains challenging due to the technical limitations of conventional detection methods, particularly their reliance on sophisticated equipment and inadequate sensitivity for *gE* gene identification in resource-limited settings. To address these critical needs, we established a novel nucleic acid detection platform that synergistically integrates recombinase-aided amplification (RAA) with CRISPR-Cas12a technology. Through systematic optimization of four CRISPR RNAs (crRNAs) and corresponding primer sets targeting conserved regions of the PRV *gE* gene, validated by fluorescence quantification and electrophoretic analysis, we developed a rapid detection system capable of achieving 10 copies/μL sensitivity within 45 min under isothermal conditions (37 °C). Clinical validation demonstrated complete diagnostic concordance with standard PCR methods, successfully identifying all 11 positive specimens from 30 clinical samples. The platform’s technical innovation lies in its sequential reaction activation mechanism that enables single-tube operation, effectively eliminating aerosol contamination risks while maintaining reaction efficiency. Detection outcomes can be interpreted through dual modalities—real-time fluorescence monitoring for quantitative analysis and lateral flow strips for visual readouts significantly enhancing field applicability. Notably, this system exhibits a 1,000-fold sensitivity improvement compared to conventional PCR, establishing itself as a robust solution for point-of-care PRV monitoring with particular utility in veterinary settings lacking advanced laboratory infrastructure.

## 1 Introduction

Pseudorabies virus (PRV) is an alphaherpesvirus closely related to herpes simplex virus type 1 (HSV-1) (Fischer et al., 2000). First documented in bovine cases in the United States in 1813 (Hanson, 1954), its etiological agent was conclusively identified by Hungarian scientist Aladár Aujeszky in 1902 through experimental inoculations in rabbits, which developed acute neurological symptoms and died within 72 h (Kohler and Kohler, 2003). The virus exhibits broad host tropism, infecting swine, ruminants, carnivores, rodents, and avian species, with ruminants developing rabies-like neurological symptoms that initially led to its designation as “pseudorabies” (Andries et al., 1978). Classified by the World Organisation for Animal Health (WOAH) as a notifiable Class B multispecies disease, PRV poses significant economic threats to global livestock production due to its high transmissibility across domestic and wild animal populations.

In China, the intensification of swine production systems coupled with increased international livestock trade since the 21st has exacerbated PRV epidemiological complexity. Suboptimal biosecurity measures and inconsistent vaccination protocols have contributed to recurrent outbreaks, resulting in substantial economic losses within the livestock sector. While molecular detection platforms—including conventional PCR, real-time PCR (RT-PCR), nanoparticle-assisted PCR (nanoPCR), recombinase-aided amplification (RAA), and loop-mediated isothermal amplification (LAMP) (Zanella et al., 2012; Ma et al., 2013; Tu et al., 2022; Zhang et al., 2010), have improved diagnostic capabilities, their dependence on specialized equipment and controlled laboratory environments severely limits deployment in resource-limited settings, particularly in rural or remote areas. RAA technology can also perform visual detection at 37 °C–42 °C. Therefore, it is suitable for promotion and use in rural or remote areas.

The CRISPR was first identified in *Escherichia coli* in 1987 (Makarova et al., 2015), representing a unique adaptive immune mechanism evolved by archaea and bacteria during their evolutionary history (Paul and Montoya, 2020). In 2016, the CRISPR-Cas system was first employed for the purpose of nucleic acid detection in molecular diagnostics. A research team (East-Seletsky et al., 2016) discovered that the Cas13 protein exhibits nonspecific collateral RNA cleavage activity. In the same year, another study (Abudayyeh et al., 2016) demonstrated that this nonspecific cleavage property of Cas13 could be harnessed for nucleic acid detection. Subsequent work (Gootenberg et al., 2017) further developed a CRISPR/Cas13-based diagnostic technology. The CRISPR-Cas revolution has since expanded into molecular diagnostics, displacing PCR in numerous applications. Cas12a, a Class 2 Type V effector protein in the CRISPR system, has been shown to recognise thymine-rich target DNA sequences guided by CRISPR RNA (crRNA) by binding to the protospacer adjacent motif (PAM) TTTN (Paul and Montoya, 2020). Upon cleavage of the target double-stranded DNA at specific sites, its trans-cleavage activity is activated, thereby enabling efficient degradation of single-stranded DNA within the system (He et al., 2024).

**Figure 1:**
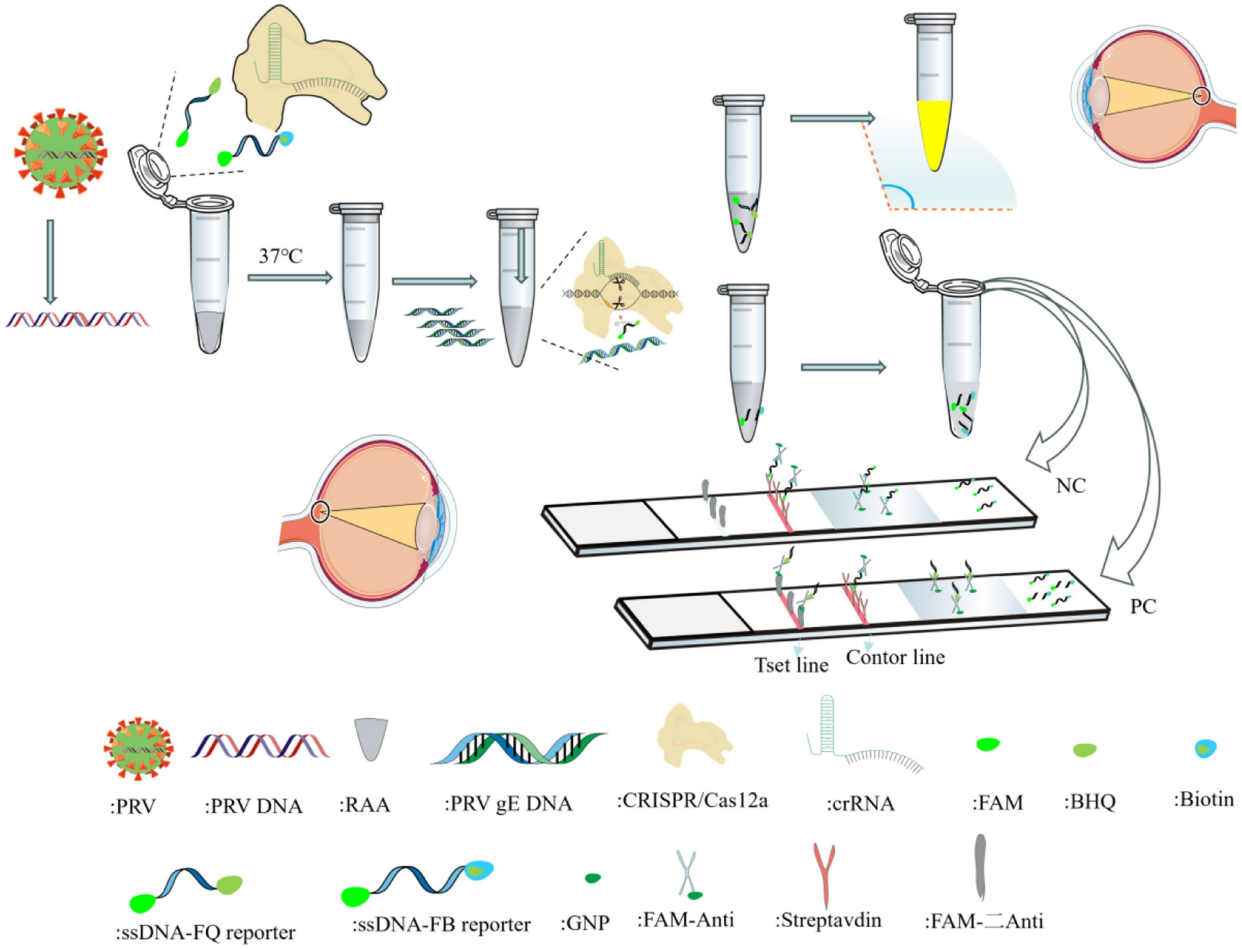
Schematic of RAA-CRISPR/Cas12a detection platform for PRV *gE* gene.

RAA is an isothermal rapid amplification method that enables efficient target DNA replication at room temperature via the synergistic action of recombinase, single-strand binding protein, and DNA polymerase (Juma et al., 2023). Compared with traditional PCR/RT-PCR requiring repeated thermal cycling (resulting in prolonged amplification) (Gleerup et al., 2025), and LAMP isothermal amplification with limitations like complex primer design and strict 65 °C reaction requirements (Zhang et al., 2010), RAA demonstrates significant advantages. Notably, although RPA shares similar principles and operates under isothermal conditions, it suffers from higher reagent costs and narrower optimal temperature ranges. When the Cas12a-crRNA complex binds target DNA by the Cas12a-crRNA ribonucleoprotein complex, the collateral cleavage activity of Cas12a is activated, leading to nonspecific degradation of reporter DNA molecules in the reaction system. Detection results can be visualized through multiple readout modalities, including fluorescence detection under UV/blue light excitation and colorimetric interpretation via lateral flow strips (Figure 1).

In this study, we developed a nucleic acid detection method targeting the *gE* gene by integrating RAA with the CRISPR/Cas12a platform. This approach demonstrates simplicity, rapidity, high sensitivity, and specificity, providing a novel solution for early-stage and field-deployable detection of pseudorabies virus.

## 2 Materials and methods

### 2.1 Viruses and sample

PRV, PEDV and TGEV were donated by the Key Laboratory of Tropical Subtropical Animal Viral Diseases (KLATSAVD). PRRSV, PCV2, CSFV and PPV positive samples (confirmed positive by laboratory testing and sequencing) were retained by our laboratory.

Clinical blood samples (*n* = 30) were collected from pigs with suspected PRV infections across 3 farms in Yunnan Province, China, during 2023–2024.

### 2.2 Reagent and primer design and synthesis

The RAA Basic Nucleic Acid Amplification Kit enables rapid isothermal amplification of target DNA at 37 °C, while its companion RAA Basic Nucleic Acid Amplification Kit (Colloidal Gold) facilitates visual detection of amplified products—both kits were procured from AmpFuture Biotechnology Co., Ltd. And the CRISPR-Cas12a-specific nucleic acid detection strips (BaoYing TongHui Biotechnology Co., Ltd., Beijing, China) were employed for visual interpretation of nucleic acid detection results following CRISPR-Cas12a system activation. The *gE* sequence of porcine pseudorabies strain (PRV-XD-F3) was compared with the PRV *gE* sequences of GenBank numbered OR161244.1, OR161238.1, OR161226.1, OR161208.1, ON261936.1, and OR161232.1 in NCBI, and the PRV *gE* sequences were designed by using primer5.0 software. The primers and crRNAs were synthesized by General Biological Co.

### 2.3 Target gene purification

The PRV *gE* gene PCR products were purified using the Universal DNA Purification Kit (Tiangen Biotech) following manufacturer’s instructions. Briefly, target bands were excised from 1% agarose gels, dissolved in B2 buffer, and purified through column adsorption. After washing steps, DNA was eluted in 15–40 μL buffer and stored at −20 °C for downstream applications.

### 2.4 Target gene cloning and transformation

The DNA product obtained was ligated into the pMD18-T vector. The ligation product was transformed into DH5a receptor cells, added to 890 mL of SOC medium and incubated for 60 min at 37 °C with shaking and cultured on L-agar medium containing X-Gal, IPTG and Amp. After colony growth, white colonies were selected with a small pipette tip and added to LB medium and incubated overnight on a shaker.

### 2.5 Positive clone validation

The bacterial solution was cultured overnight, DNA was extracted, and the PCR amplification system and procedure (target fragment amplification, recovery, and purification) was used. The PCR products were detected by 1% agarose gel electrophoresis, and the size of the bands was observed to determine whether the transformation was successful or not.

### 2.6 Recombinant plasmid preparation

Recombinant plasmids were extracted using the Plasmid Extraction Kit following the manufacturer’s protocol. Briefly, bacterial cultures were pelleted, lysed with Buffers P1, P2, and P3, and centrifuged to remove debris. The supernatant was purified through an adsorption column, washed, and eluted in 50–100 μL elution buffer. Plasmid concentration was determined spectrophotometrically, and samples were sent for sequencing (Sangon Biotech (Shanghai) Co., Ltd.).

### 2.7 Calculation of recombinant plasmid concentration

The concentration of validated recombinant plasmid was determined using a nucleic acid concentration meter. The average molecular weight of each base is 660. The pMD18-T-PRV-*gE* positive plasmid contains 4,500 bases, and the molecular weight of a single plasmid is 660 × (pMD18-T vector length + insertion gene length), and the final copy number is calculated according to the following formula (Kong, 2021).


$$\begin{array}{l} \mathrm{DNA}\;\text{copy number}/\mathrm{\mu L}\\ =\frac{\left[6.02\times 1,023\times \text{concentration}\;(\mathrm{ng}/\mathrm{\mu L})\times 10^{-9}\right]}{\left[(\mathrm{pMD}18‐T\;\text{Vector length}+\text{insertion gene length})\times 660\right]} \end{array}$$

### 2.8 crRNA and primer design and preparation

Referring to the selection requirements of Cas12a protein PAM and protospacer sequences, ssDNA sequences were analyzed and crRNAs were designed using the CRISPR-DT online website,^1^ i.e., TTTN sequences were identified within the highly conserved *gE* target region of PRV, and 23 nt was selected as the 23 bp target sequence.

Based on the determination and screening of crRNA recognition sequences, RAA primers were designed in their upstream and downstream regions by applying Primer5.0 software, respectively. Primer lengths were optimized for maximal amplification efficiency under the selected RAA conditions while maintaining target specificity.

The complete primer sequences and characterization are shown in Table 1.

**Table 1:** Primer and probe sequences.

Name	Sequence (5′–3′)
crRNA1	UAAUUUCUACUAAGUGUAGAUCCGCCACGCUGGACUGGUACU
crRNA2	UAAUUUCUACUAAGUGUAGAUUGCUGGCGCUGGGCUCCUUCGUG
crRNA3	UAAUUUCUACUAAGUGUAGAUUCCGGAUCGCGGAACCAGACGUC
crRNA4	UAAUUUCUACUAAGUGUAGAUAGCGGGGCGGGACAUCAACAGGC
RAA-F1	CGTGTTCTTTGTGGCGGTGGGC
RAA-R1	CGCGGGTGGTAGATGCAGGGCT
RAA-F2	GTCGCCGCACCTGAGCGTCCTGCGGGC
RAA-R3	TACGAGCCCTGCATCTACCACCCGCGCG
RAA-F3	CCGAGTACGTCACGGTCATCAAGGAG
RAA-R3	CCGGGAGCACAGCACGCAGAGCCAGA
RAA-F4	GCCGAGTACGTCACGGTCATCAA
RAA-R4	GGGAGCACAGCACGCAGAGCCAG
F-Q	6-FAM-TTATT-BHQ I
F-B	6-FAM-TTTTTTTATTTTTTT-Biotin
PCR-R	CATCTGGCTCTGCGTGCTGT
PCR-F	TTGGGTCCATTCGTCACTTCC

### 2.9 CRISPR/Cas12a cutting system

Enzyme-free water was used as a negative control, and 1 μL Cas12a Naclease (1 μM), 2 μL pMD18-T-PRV-*gE*, 1 μL crRNA (1 μM), 1 μL F-Q (10 μM), and 2 μL of 10 × Cas12 reaction Buffer were added to a fluorescence octuple tube, and replenished to 20 μL with enzyme-free water. Next, the quantitative PCR was performed in a PCR instrument at 37 °C for 25 min, and the fluorescence signals were observed after passing through a UV lamp and a blue light.

### 2.10 RAA amplification system

The reaction temperature was set at 37 °C to meet the requirements for RAA amplification and to maintain optimal Cas12a cleavage activity. This was verified by a preliminary experiment.

The amplification system was constructed according to the RAA isothermal amplification kit: 25 μL of bufferA, 2.5 μL of bufferB (MgOAC), 2 μL of RAA upstream primer (10 μM), 2 μL of RAA downstream primer (10 μM), 16.5 μL of enzyme-free water, and 2 μL of target DNA, for a total of 50 μL of system. The reaction system was incubated on a metal bath at 37 °C for 20 min to complete the amplification of target DNA. After the reaction, an equal volume of nucleic acid extraction reagent (saturated phenol: chloroform: isoamyl alcohol = 25:24:1) was added to the RAA amplification product; after mixing, the reaction was centrifuged at 12,000 × g for 1 min; 7 μL of the supernatant was sucked up and detected by 1.5% agar gel electrophoresis to screen for the RAA primers.

### 2.11 PCR assay

PCR primers were established in our laboratory, the primer sequence: 5′-CATCTGGCTCTCTGCGTGCTGT-3′, 5′-TTGGGTCCATTCGTCACTTCC-3′, PCR amplification was performed using the pMD18-T-PRV-*gE* positive plasmid as a template with 10-fold isocratic dilution, PCR amplification system (25 μL): 2 × Taq PCR Master Mix 12.5 μL, 1 μL of each specific primer upstream and downstream, 1 μL of each reverse transcription product, 9.5 μL of sterilized double-distilled water, 1 μL of each reverse transcription product. PCR amplification system (25 μL): 2 × Taq PCR Master Mix 12.5 μL, 1 μL of each specific primer upstream and downstream, 1 μL of each reverse transcription product, and 9.5 μL of sterilized double-distilled water to make up the reaction system. The reaction system was supplemented with 9.5 μL of sterilized double-distilled water. The reaction system was placed on a fluorescence quantitative PCR instrument and pre-denatured at 98 °C for 10 s, followed by 55 °C for 30 s, 72 °C for 2 min, and then cycling the reaction for 35 times, and then detected by 1.5% agar gel electrophoresis.

### 2.12 Statistical analysis

Data were analyzed using GraphPad Prism 9.0. Fluorescence intensity differences were assessed via one-way ANOVA with Tukey’s post-hoc test. *p <* 0.05 was considered significant.

## 3 Results

### 3.1 Standard positive plasmid construction

**Figure 2:**
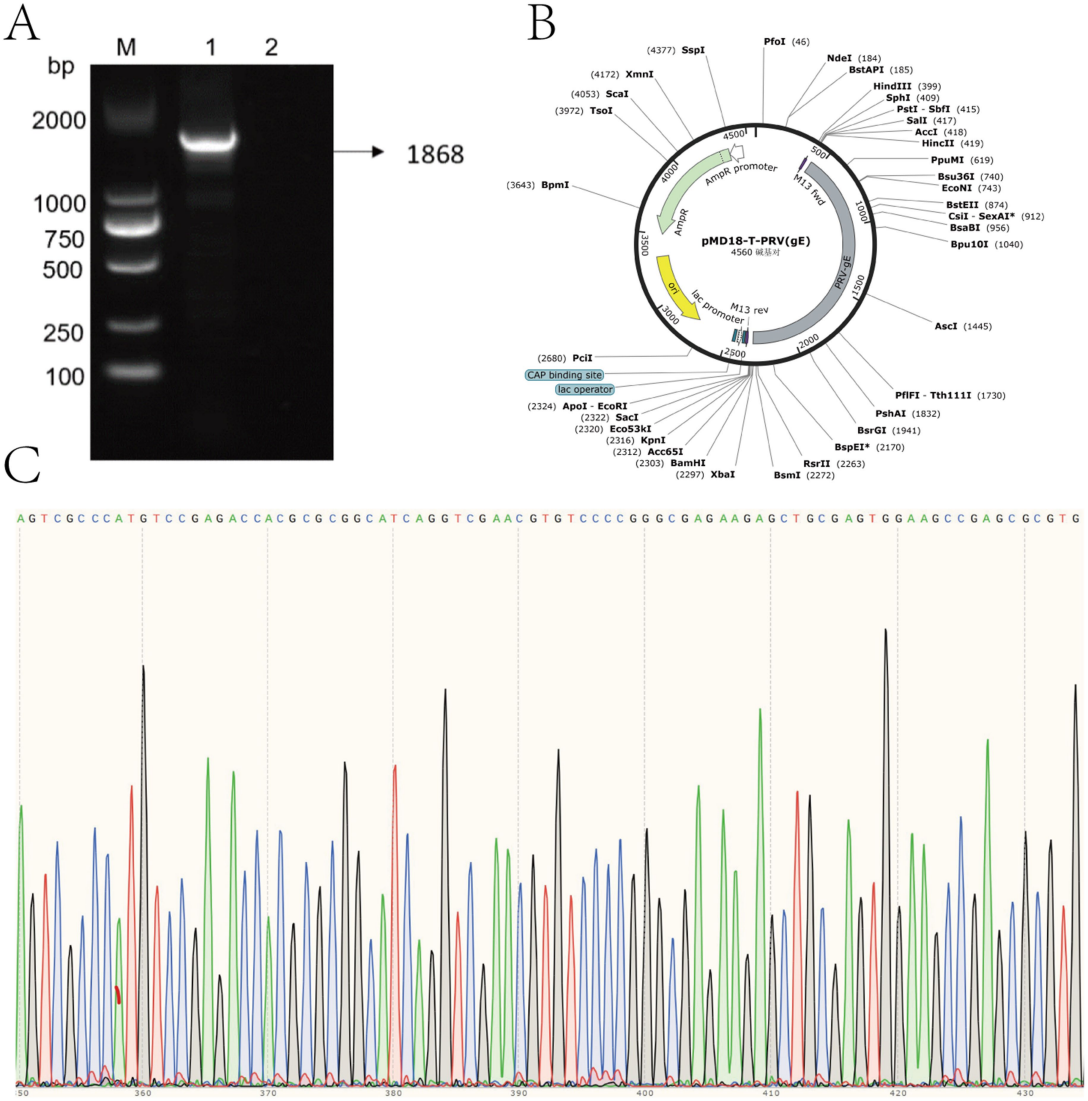
Construction and verification of recombinant pMD18-T-PRV-*gE* plasmid. **(A)** Agarose gel electrophoresis of PCR-amplified PRV *gE* gene fragment. Lane M: DNA marker (sizes indicated in bp). Lane 1: 1,868 bp target amplicon. Lane 2: Negative control. **(B)** Schematic map of constructed pMD18-T-PRV-*gE* plasmid, showing key restriction sites and inserted PRV *gE* fragment orientation. **(C)** Sanger sequencing chromatogram of the cloned insert, demonstrating 100% sequence identity with the PRV *gE* target region.

Using PRV nucleic acid as a template, the constructed recombinant plasmid standard pMD18-T-PRV-*gE* were identified by PCR, 1,868 bp had a specific target band appearing, which was consistent with the expected fragment size, and the negative did not show a band (Figure 2A). The sequencing results were compared with the sequence of *gE* of PRV-XD-F3 strain without base mutation or deletion, which proved the successful construction of pMD18-T-PRV-*gE* recombinant positive plasmid (Figure 2B-C). The standard positive plasmid was calculated as 4 × 10^10^ copies/μL according to the formula.

### 3.2 Screening of crRNA and primers

**Figure 3:**
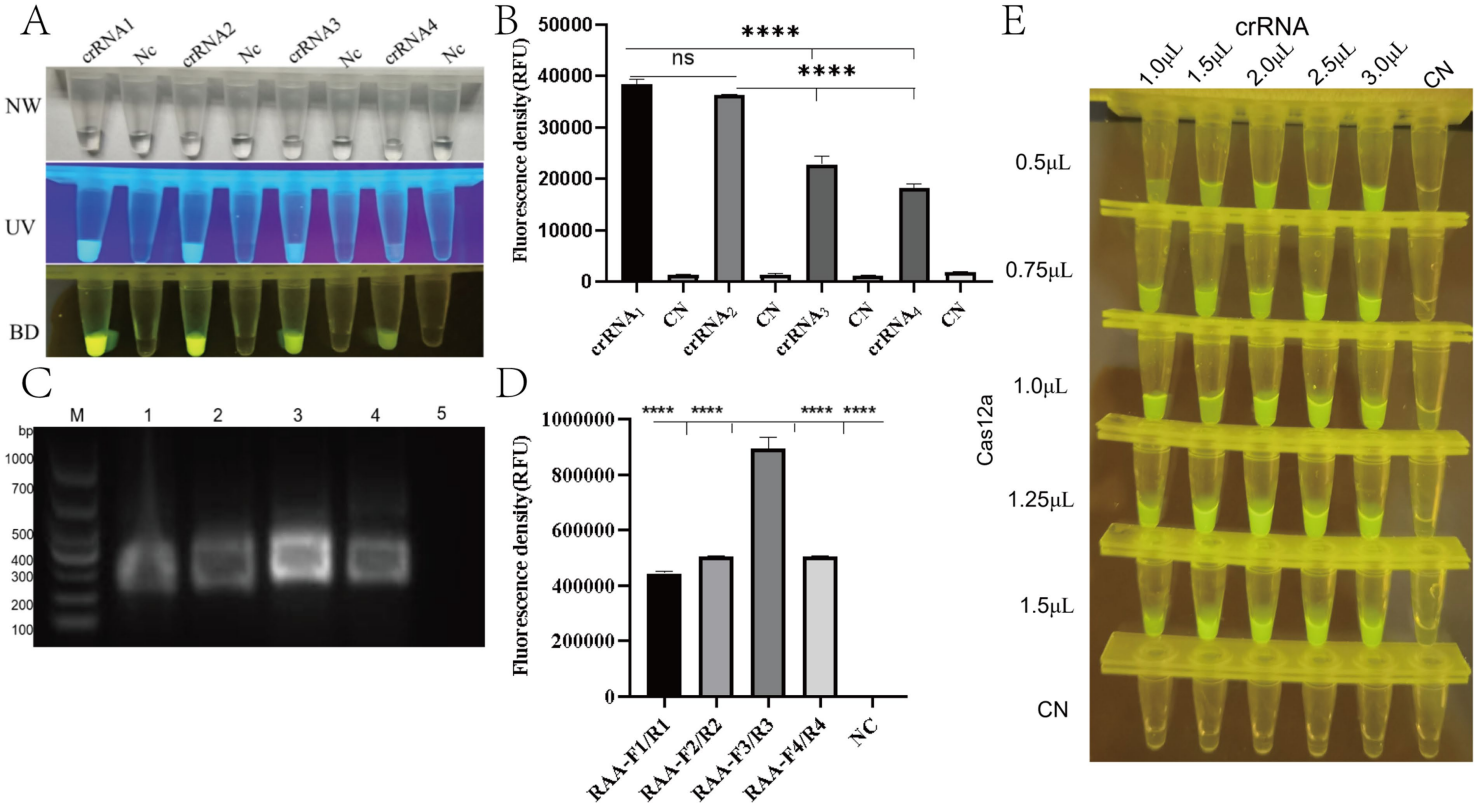
Optimization of RAA-CRISPR/Cas12a detection system for PRV *gE* gene. **(A)** Fluorescence visualization under UV (365 nm, top) and blue light (470 nm, bottom) illumination showing distinct signal intensities for different crRNA designs. Positive reactions exhibit bright green fluorescence (FAM reporter cleavage), while negative controls remain dark. **(B)** Quantitative analysis of fluorescence intensity (a.u.) for four crRNA candidates (crRNA1-4). ^****^p < 0.0001 vs. negative control (NC) by Student’s *t*-test. Error bars represent SD (*n* = 3). **(C)** Agarose gel (1.5%) electrophoresis of RAA amplification products. Lane M: DNA ladder (sizes indicated). Lanes 1–5: Amplified products from different primer sets (expected size ~200 bp). Arrow indicates target band. **(D)** Fluorescence intensity comparison of different Cas12a:crRNA molar ratios (1:1 to 3:1). Optimal activity observed at 2:1 ratio (^****^p < 0.0001). **(E)** Tube array test showing fluorescence intensity gradient with varying crRNA (50–200 nM) and Cas12a (100–400 nM) concentrations. Brightest signals observed at 200 nM crRNA + 400 nM Cas12a.

Based on the conserved region of the PRV *gE* gene, multiple sequence alignment was performed using Snap Gene software. Four crRNAs (crRNA1-4) targeting distinct PAM sites were designed via the CRISPR-DT online platform (see text footnote 1). To evaluate the trans-cleavage activity of crRNA-guided Cas12a, CRISPR/Cas12a detection systems containing individual crRNAs were constructed. Under excitation with ultraviolet (365 nm) and blue light (470 nm), all four crRNAs triggered cleavage of the fluorescent reporter molecule (FAM-TTATT-BHQ1), generating visible fluorescence signals (Figure 3A). Quantitative analysis of fluorescence intensity from blue light-transmitted images was performed Quantitative analysis of fluorescence signals demonstrated significantly higher trans-cleavage activity for crRNA1 and crRNA2 compared to crRNA3 and crRNA4 (*p* < 0.05), with no statistically significant difference observed between crRNA1 and crRNA2 (Figure 3B). For the PAM targets of crRNA1 and crRNA2, two pairs of RAA primers (RAA-F1/R1, F2/R2, F3/R3, F4/R4) were designed. All primer pairs specifically amplified target fragments. Notably, the RAA-F3/R3 primers paired with crRNA2 exhibited significantly higher amplification efficiency compared to other primers, with no detectable non-specific bands (Figure 3C). Based on these findings, crRNA2 and its corresponding RAA-F3/R3 primer pair were selected for subsequent experiments.

In order to optimise the CRISPR/Cas12a fluorescence assay, a screening procedure was performed for the concentration of Cas12a nuclease and crRNA. The experimental results demonstrated that the CRISPR/Cas12a detection system exhibited optimal target recognition and the strongest fluorescence signal when using 1.0 μL of Cas12a protein (1 μM) and 2.0 μL of crRNA (1 μM), corresponding to a crRNA:Cas12a molar ratio of 2:1 (Figure 3D-E).

### 3.3 RAA-CRISPR/Cas12a assay system establishment

The RAA-CRISPR/Cas12a detection system was established by optimizing the reaction components based on the manufacturer’s protocol.

**Figure 4:**
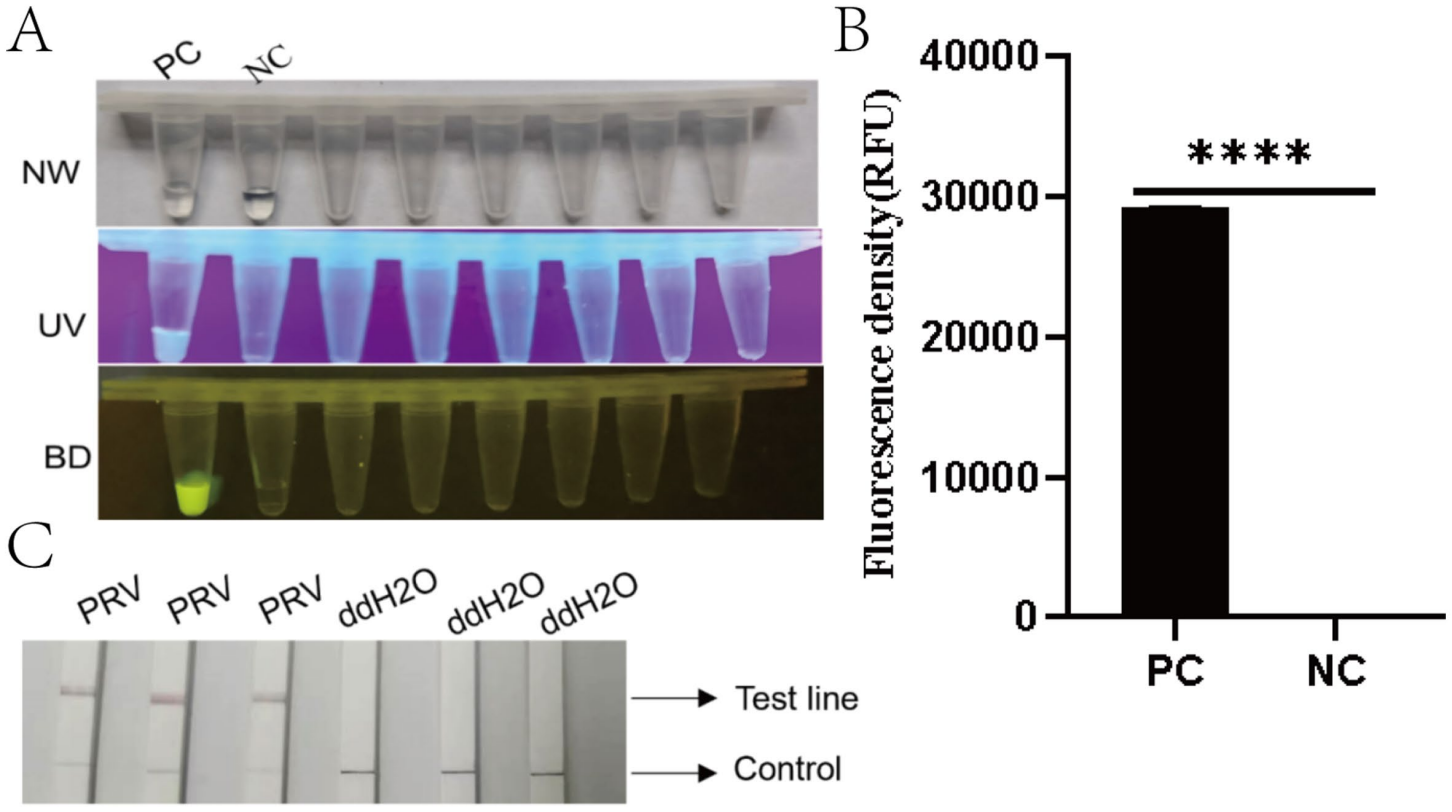
Performance evaluation of the RAA-CRISPR/Cas12a detection system. **(A)** Three-view microplate imaging: NW (natural light), UV (ultraviolet light at 365 nm), and BD (bioluminescence detection). PC (positive control) wells exhibit bright yellow fluorescence in BD view (arrow), while NC (negative control) wells remain dark. **(B)** Quantitative fluorescence density analysis (RFU) showing significantly higher signal in PC versus NC (^****^p < 0.0001). Error bars represent SD (*n* = 3 technical replicates). **(C)** Lateral flow strip results: Three PRV-positive samples show both control (C, upper) and test (T, lower) lines (red arrows), while ddH_2_O negative controls display only C lines. Strip images were captured at 10 min post-immersion.

Fluorescence signals were observed using probe F-Q under UV light (365 nm) and blue light (470 nm). Positive samples exhibited strong fluorescence, whereas negative controls showed no fluorescence (Figures 4A,B). A statistically significant difference was observed between the test system and negative control groups (^****^p < 0.0001). For probe F-B, positive samples displayed distinct bands at both the quality control (C) and detection (T) lines, while negative controls (nuclease-free water) showed only the C line (Figure 4C). These results aligned with the expected outcomes.

### 3.4 Specificity evaluation

**Figure 5:**
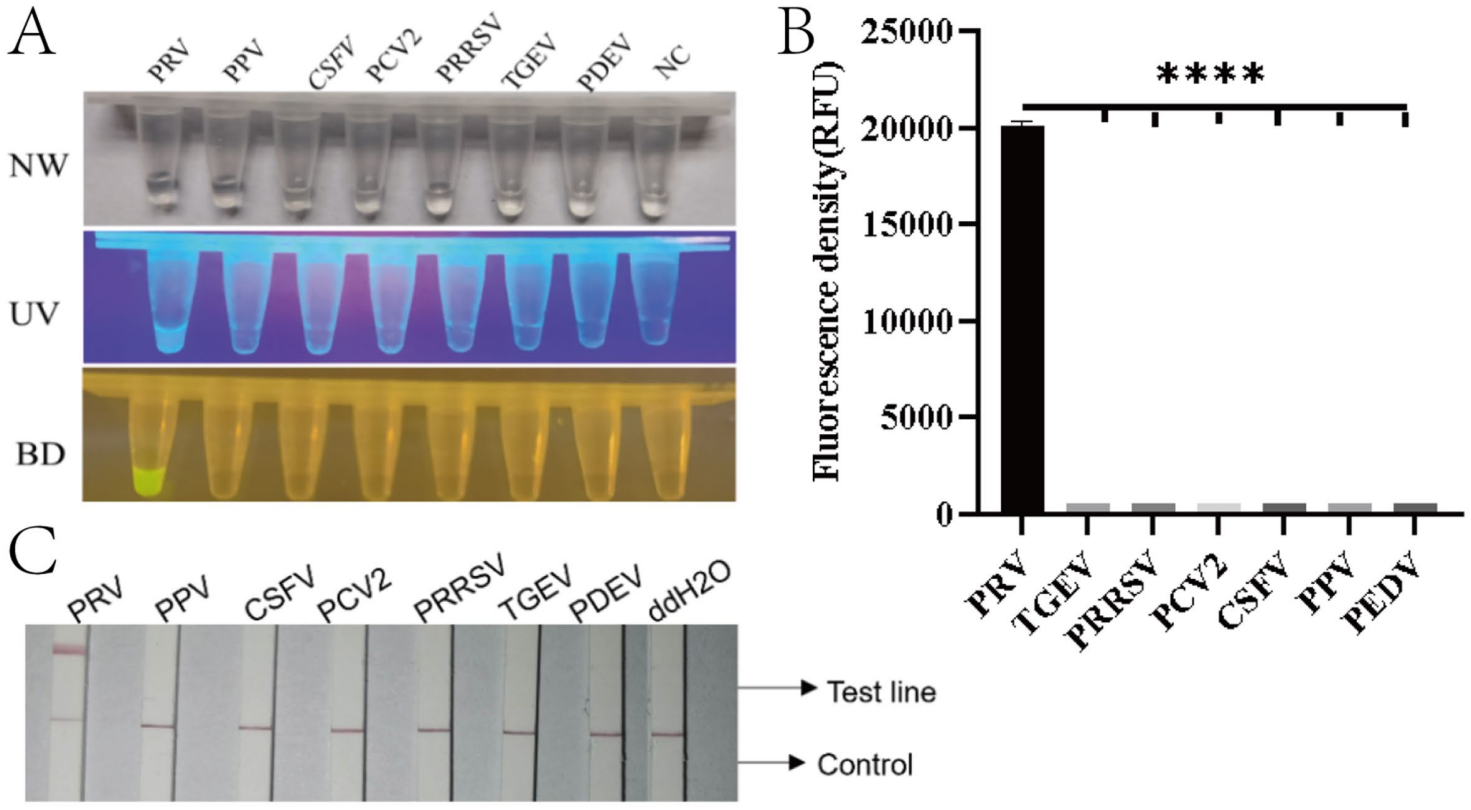
Specificity evaluation of the RAA-CRISPR/Cas12a detection system for PRV. **(A)** Three-view detection results showing specific recognition of PRV. From left to right: NW (natural light), UV (365 nm excitation), and BD (blue light detection with 470 nm filter) images. Only PRV samples exhibit bright yellow fluorescence in BD view (arrow), while other porcine viruses (PPV, PRRSV, PCV2, CSFV, PEDV, TGEV) and negative control (NC, ddH_2_O) remain non-fluorescent. **(B)** Quantitative fluorescence intensity analysis (RFU) demonstrating exclusive detection of PRV (^****^p < 0.0001). Error bars represent SD of triplicate experiments. **(C)** Lateral flow assay results validating specificity: Only PRV-infected samples develop both control (C) and test (T) lines (red arrows), while other pathogens and negative controls show single C lines. Strips were photographed at 10 min after sample application.

To evaluate the specificity of the method, seven common porcine viral nucleic acids (PRV, PEDV, TGEV, PRRSV, PCV2, CSFV, and PPV) along with nuclease-free water were tested as negative controls. Fluorescence analysis revealed significant signals exclusively in the PRV-positive group (Figures 5A,B). Lateral flow strip results showed that only PRV samples generated both control (C) and test (T) lines, while other viral nucleic acids (PEDV, TGEV, PRRSV, PCV2, CSFV, PPV) and the negative control displayed only the C line (Figure 5C). These findings demonstrate that the RAA-CRISPR/Cas12a system exhibits high specificity without cross-reactivity with the genomic DNA of the other six pathogens tested.

### 3.5 Sensitivity evaluation

**Figure 6:**
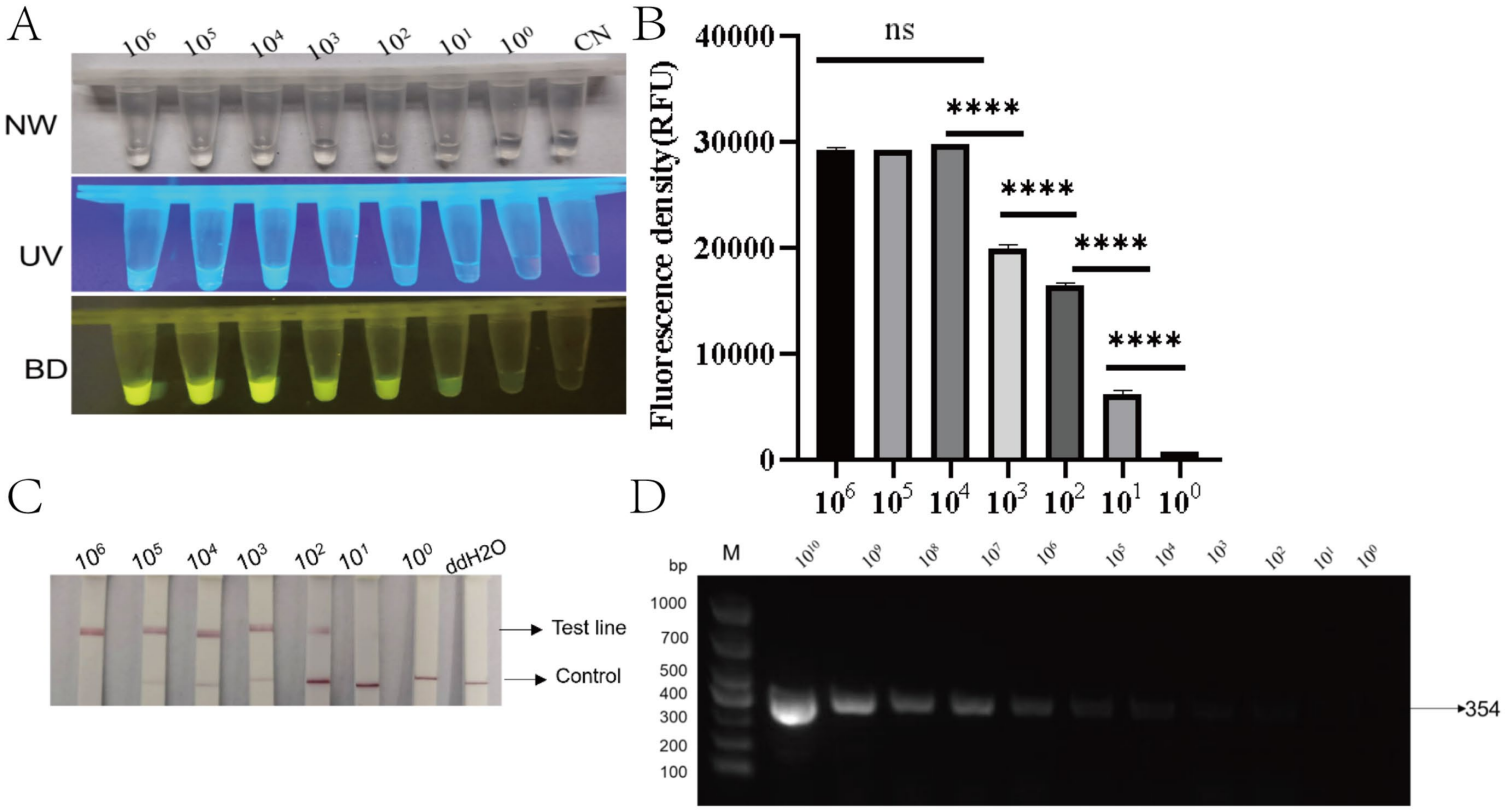
Sensitivity analysis of the RAA-CRISPR/Cas12a detection system. **(A)** Three-mode detection of serial dilutions (10⁶–10^0^ copies/μL) showing: NW (natural light), UV (365 nm excitation), and BD (bioluminescence detection) views. Fluorescence intensity decreases proportionally with target concentration. CN: negative control. **(B)** Quantitative fluorescence intensity (RFU) demonstrating logarithmic correlation with target concentration. ^****^p < 0.0001 for 10¹–10⁶ vs. NC; ns, not significant (10⁰ vs. NC) (*n* = 3). **(C)** Lateral flow strips corresponding to concentrations in **(A)**. Visible test lines (T) appear down to 10² copies/μL (faint at 10¹). C: control line; ddH₂O: negative control. **(D)** Agarose gel electrophoresis confirming specific 354 bp amplicons (arrow) across all detectable concentrations. M: DNA ladder (sizes in bp).

To evaluate the sensitivity of the RAA-CRISPR/Cas12a system for PRV detection, a 10-fold serially diluted standard plasmid (1 × 10^6^ to 1 × 10^0^ copies/μL) was analyzed alongside nuclease-free water as the negative control. Fluorescence signals under blue light illumination were clearly detectable at a template concentration of 10 copies/μL (Figure 6A). Statistical analysis confirmed significant differences in fluorescence intensity between 10 copies/μL samples and negative controls (^****^p < 0.001, Figure 6B), establishing the detection limit of the RAA-CRISPR/Cas12a fluorescence system at 10 copies/μL. Lateral flow strip analysis revealed distinct test line (T) bands for templates ranging from 10^6^ to 10^2^ copies/μL. Faint but discernible T-line bands were observed at 10 copies/μL, while no bands appeared in negative controls (Figure 6C). In contrast, conventional PCR exhibited a detection limit of 1 × 10^4^ copies/μL, producing expected 354 bp amplicons only above this threshold (Figure 6D). These data demonstrate that the RAA-CRISPR/Cas12a system achieves 1,000-fold greater sensitivity than conventional PCR, confirming its high analytical performance for PRV detection.

### 3.6 Clinical sample validation

**Figure 7:**
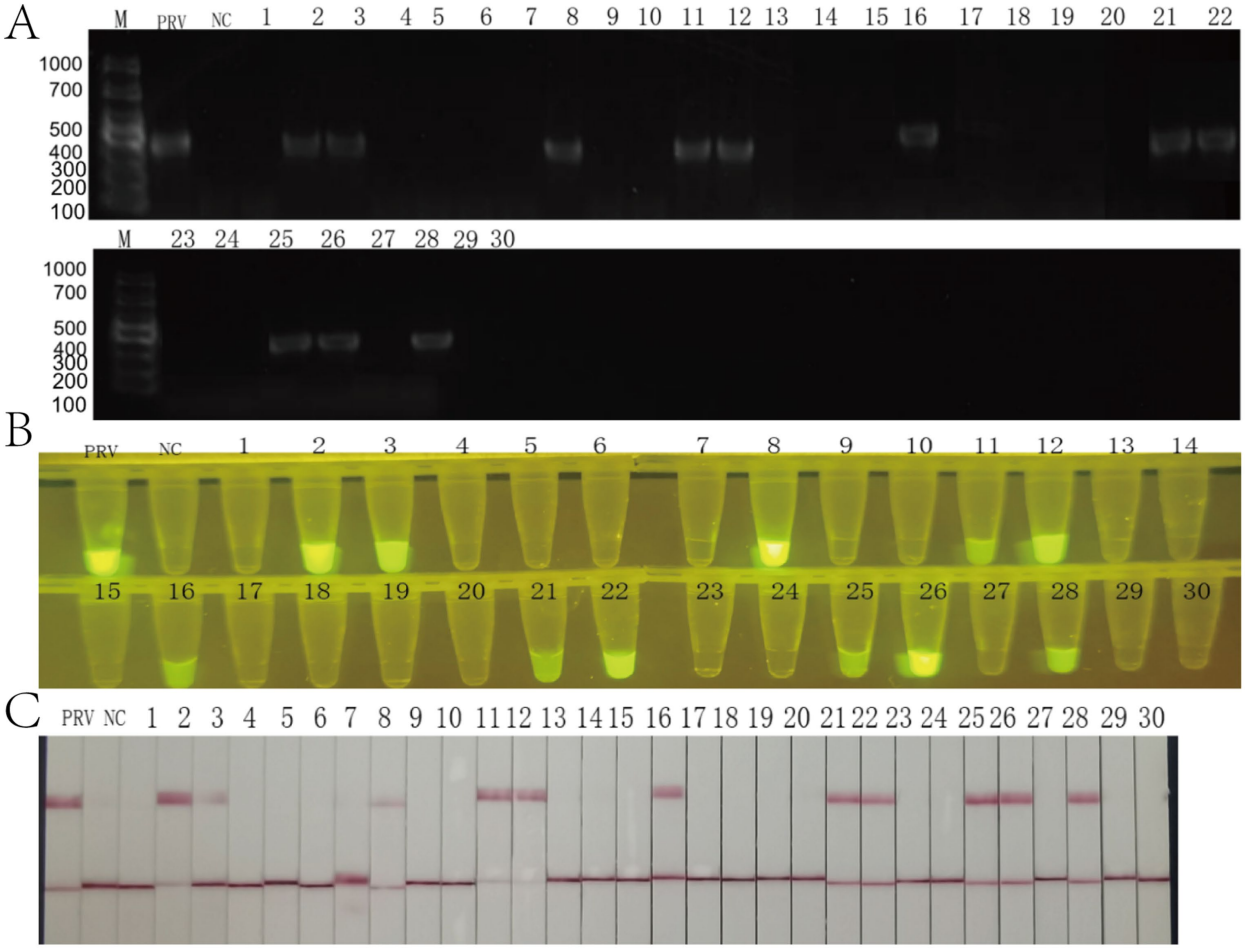
Clinical validation of the RAA-CRISPR/Cas12a detection system using 30 field samples. **(A)** Agarose gel electrophoresis (1.5%) of conventional PCR products. Lane M: DNA ladder (sizes in bp indicated). PRV: positive control (354 bp amplicon, arrow). NC: negative control. Lanes 1–30: clinical samples (11/30 positive). **(B)** Fluorescence detection under blue light (470 nm). PRV: strong positive control. NC: negative control. Samples 2, 3, 4, 7, 24, 26 show bright fluorescence (yellow arrows), corresponding to PCR positives. **(C)** Lateral flow strip results. PRV: positive control (C and T lines visible). NC: negative control (C line only). Clinical samples display 100% concordance with PCR.

A total of 30 blood samples were collected from clinical samples of a breeding farm in Yunnan Province, and nucleic acid extraction was performed, and RAA-CRISPR/Cas12a fluorescence, test strip method and PCR were compared with nucleic acid as the detection template. All three detection methods demonstrated identical positive rates of 36.7% (11/30) across the 30 clinical samples: conventional PCR (Figure 7A), RAA-CRISPR/Cas12a fluorescence assay (Figure 7B), and lateral flow strip analysis (Figure 7C). Comparative validation revealed 100% concordance between both CRISPR-based methods and PCR in sensitivity and specificity metrics. The results showed that the RAA-CRISPR/Cas12a fluorescence method, the test strip method, and all of the RAA-CRISPR/Cas12a fluorescence methods established in this study can be applied to the rapid clinical testing.

## 4 Discussion

The RAA-CRISPR/Cas12a system developed in this study combines the advantages of rapid isothermal amplification with the high specificity of CRISPR-based detection. Compared to conventional PCR and RT-PCR, which rely on thermal cycling and prolonged amplification times (Ding et al., 2020). RAA offers simplified operation, faster results, and compatibility with isothermal conditions, eliminating the need for expensive thermocyclers. While LAMP is another isothermal alternative, its complex primer design, multi-step amplification process, and requirement for 65 °C incubation limit its practicality (Zhang et al., 2010). RPA isothermal amplification works in a similar way to RAA, but is more costly and has a smaller optimal temperature range for RPA. The use of optimized Cas12a dosage and probe concentration resulted in reduced reagent costs compared to those of conventional CRISPR detection systems.

In this study, the isothermal amplification technology RAA was effectively combined with the CRISPR/Cas12a system, and multiple crRNA and RAA isothermal amplification primers were designed, in which RAA was amplified in order to obtain a large number of target genes to ensure the sensitivity of the assay. crRNA specifically binds to the target sequences in the RAA amplification product for the cleavage reaction, and this dual-specific base complementary binding makes the assay provide detection of high specificity, maximizing the advantages of each. We established two detection platforms: (1) a fluorescence-based RAA-CRISPR/Cas12a assay using reporter probes, and (2) a lateral flow assay employing biotin-labeled probes for visual interpretation. Both methods demonstrated excellent specificity and sensitivity, achieving a detection limit of 10 copies/μL. While the RAA-CRISPR/Cas12a system has been previously validated for PCV3, ASFV, and PPRSV detection, this study represents its first application for PRV identification.

The RAA-CRISPER/Cas12a fluorescence, test strip detection method established in this study, in the detection process RAA amplification reagents only need to be used to 1/5 of the original RAA amplification system to meet the efficiency of nucleic acid amplification, greatly reducing the cost of the reaction (Yang et al., 2024). Specific sequence recognition, crRNA with about 23 short nucleotides was designed to combine with RAA specific amplification to make it double specificity, which greatly increased the accuracy of detection (Liu et al., 2022). In order to solve the problem that RAA amplification system and CRISPR/Cas12a system cannot effectively coexist and RAA open cap is prone to aerosol contamination, the innovative CRISPR/Cas12a system was dropped on the inner cap of the PCR tube, while the RAA amplification system was placed on the bottom of the PCR tube, and the RAA amplification reaction was finished, and then the CRISPER/Cas12a shear system was centrifuged to the bottom of the tube. Cas12a protein starts to recognize the target gene and activate the cut reporter. The whole process is carried out in one reaction tube, which simplifies the steps and reduces the possibility of false positives, but there are still some improvements to be made in the future clinical application of this method, such as the CRISPR/Cas12a components tend to slip out of the cap when the cap of the PCR tube is closed (Ling et al., 2021). In the future, we will try to develop an isolation system, using liquid paraffin and solid paraffin to realize the solid state at room temperature and liquid state at 37 °C, and isolate the CRISPR/Cas12a system in the inner lid of the PCR tube with the isolation system, and then centrifuge the CRISPR/Cas12a system and the RAA amplification product after RAA amplification at 37 °C, which will greatly facilitate the clinical detection. While our crRNA target site shows high conservation across sequenced PRV strains, future studies should empirically validate detection performance against geographically diverse isolates, particularly those with known *gE* polymorphisms. Although this study’s clinical validation showed complete agreement with the PCR assay, subsequent validation using a larger sample size in a different geographic region would help further assess this method’s robustness.

## 5 Conclusion

This study successfully established on-site rapid nucleic acid detection methods for Pseudorabies virus, both applicable for clinical PRV detection. The RAA-CRISPR/Cas12a fluorescence assay and the RAA-CRISPR/Cas12a lateral flow strip assay demonstrated a detection limit of 10 copies/μL for PRV, exhibiting excellent sensitivity and specificity. These methods hold promising application prospects for rapid and accurate pathogen identification in field or clinical settings.

## Data Availability

The original contributions presented in the study are included in the article/supplementary material, further inquiries can be directed to the corresponding author.

## Footnotes

^1^ http://crispor.tefor.net/

## References

[ref1] Abudayyeh O. O. Gootenberg J. S. Konermann S. Joung J. Slaymaker I. M. Cox D. B. T.. (2016). C2c2 is a single-component programmable RNA-guided RNA-targeting CRISPR effector. Science 353:aaf5573. doi: 10.1126/science.aaf5573, PMID: 27256883 PMC5127784

[ref2] Andries K. Pensaert M. B. Vandeputte J. (1978). Effect of experimental infection with pseudorabies (Aujeszky’s disease) virus on pigs with maternal immunity from vaccinated sows. Am. J. Vet. Res. 39, 1282–1285. 211882

[ref3] Ding G. Fu Y. Li B. Chen J. Wang J. Yin B.. (2020). Development of a multiplex RT-PCR for the detection of major diarrhoeal viruses in pig herds in China. Transbound. Emerg. Dis. 67, 678–685. doi: 10.1111/tbed.13385, PMID: 31597013 PMC7168528

[ref4] East-Seletsky A. O’Connell M. R. Knight S. C. Burstein D. Cate J. H. Tjian R.. (2016). Two distinct RNase activities of CRISPR-C2c2 enable guide-RNA processing and RNA detection. Nature 538, 270–273. doi: 10.1038/nature19802, PMID: 27669025 PMC5576363

[ref5] Fischer T. Buttner M. Rziha H. J. (2000). T helper 1-type cytokine transcription in peripheral blood mononuclear cells of pseudorabies virus (Suid herpesvirus 1)-primed swine indicates efficient immunization. Immunology 101, 378–387. doi: 10.1046/j.1365-2567.2000.00124.x, PMID: 11106942 PMC2327083

[ref6] Gleerup D. Trypsteen W. Fraley S. I. de Spiegelaere W. (2025). Digital PCR in virology: current applications and future perspectives. Mol. Diagn. Ther. 29, 43–54. doi: 10.1007/s40291-024-00751-9, PMID: 39487879

[ref7] Gootenberg J. S. Abudayyeh O. O. Lee J. W. Essletzbichler P. Dy A. J. Joung J.. (2017). Nucleic acid detection with CRISPR-Cas13a/C2c2. Science 356, 438–442. doi: 10.1126/science.aam9321, PMID: 28408723 PMC5526198

[ref8] Hanson R. P. (1954). The history of pseudorabies in the United States. J. Am. Vet. Med. Assoc. 124, 259–261. 13142964

[ref9] He J. Hu X. Weng X. Wang H. Yu J. Jiang T.. (2024). Efficient, specific and direct detection of double-stranded DNA targets using Cas12f1 nucleases and engineered guide RNAs. Biosens. Bioelectron. 260:116428. doi: 10.1016/j.bios.2024.116428, PMID: 38805891

[ref10] Juma K. M. Inoue E. Asada K. Fukuda W. Morimoto K. Yamagata M.. (2023). Recombinase polymerase amplification using novel thermostable strand-displacing DNA polymerases from *Aeribacillus pallidus* and *Geobacillus zalihae*. J. Biosci. Bioeng. 135, 282–290. doi: 10.1016/j.jbiosc.2023.01.009, PMID: 36806411

[ref11] Kohler M. Kohler W. (2003). Zentralblatt für Bakteriologie—100 years ago Aladár aujeszky detects a ‘new’ disease—or: it was the cow and not the sow. Int. J. Med. Microbiol. 292, 423–427. doi: 10.1078/1438-4221-00233 12635925

[ref12] Kong K. K. (2021). “Development and evaluation of rapid and low-cost detection system for apple groove virus based on Crispr-Cas12a technology” in Master thesis (Zhengzhou: Henan Agricultural University).

[ref13] Ling X. Chang L. Chen H. Gao X. Yin J. Zuo Y.. (2021). Improving the efficiency of CRISPR-Cas12a-based genome editing with site-specific covalent Cas12a-crRNA conjugates. Mol. Cell 81, 4747–4756.e7. doi: 10.1016/j.molcel.2021.09.021, PMID: 34648747

[ref14] Liu Y. Pinto F. Wan X. Yang Z. Peng S. Li M.. (2022). Reprogrammed tracrRNAs enable repurposing of RNAs as crRNAs and sequence-specific RNA biosensors. Nat. Commun. 13:1937. doi: 10.1038/s41467-022-29604-x, PMID: 35410423 PMC9001733

[ref15] Ma X. Cui Y. Qiu Z. Zhang B. Cui S. (2013). A nanoparticle-assisted PCR assay to improve the sensitivity for rapid detection and differentiation of wild-type pseudorabies virus and gene-deleted vaccine strains. J. Virol. Methods 193, 374–378. doi: 10.1016/j.jviromet.2013.07.018, PMID: 23872268

[ref16] Makarova K. S. Wolf Y. I. Alkhnbashi O. S. Costa F. Shah S. A. Saunders S. J.. (2015). An updated evolutionary classification of CRISPR-Cas systems. Nat. Rev. Microbiol. 13, 722–736. doi: 10.1038/nrmicro3569, PMID: 26411297 PMC5426118

[ref17] Paul B. Montoya G. (2020). CRISPR-Cas12a: functional overview and applications. Biom. J. 43, 8–17. doi: 10.1016/j.bj.2019.10.005, PMID: 32200959 PMC7090318

[ref18] Tu F. Zhang Y. Xu S. Yang X. Zhou L. Ge X.. (2022). Detection of pseudorabies virus with a real-time recombinase-aided amplification assay. Transbound. Emerg. Dis. 69, 2266–2274. doi: 10.1111/tbed.14241, PMID: 34273259

[ref19] Yang Y. Kong X. Yang J. Xue J. Niu B. Chen Q. (2024). Rapid nucleic acid detection of *Listeria monocytogenes* based on RAA-CRISPR Cas12a system. Int. J. Mol. Sci. 25:3477. doi: 10.3390/ijms25063477, PMID: 38542449 PMC10971093

[ref20] Zanella E. L. Miller L. C. Lager K. M. Bigelow T. T. (2012). Evaluation of a real-time polymerase chain reaction assay for Pseudorabies virus surveillance purposes. J. Vet. Diagn. Invest. 24, 739–745. doi: 10.1177/1040638712447279, PMID: 22621947

[ref21] Zhang C. Cui S. Zhu C. (2010). Loop-mediated isothermal amplification for rapid detection and differentiation of wild-type pseudorabies and gene-deleted virus vaccines. J. Virol. Methods 169, 239–243. doi: 10.1016/j.jviromet.2010.07.034, PMID: 20691214 PMC7112886

